# Trial design: Computer guided normal-low versus normal-high potassium control in critically ill patients: Rationale of the GRIP-COMPASS study

**DOI:** 10.1186/1471-2253-10-23

**Published:** 2010-12-31

**Authors:** Miriam Hoekstra, Mathijs Vogelzang, Iwan CC van der Horst, Annemieke Oude Lansink, Joost MAA van der Maaten, Farouq Ismael, Felix Zijlstra, Maarten WN Nijsten

**Affiliations:** 1 Department of Anesthesiology, University Medical Center Groningen, University of Groningen, Hanzeplein 1, 9700 RB, Groningen, the Netherlands; 2 Department of Cardiology, University Medical Center Groningen, University of Groningen, Hanzeplein 1, 9700 RB, Groningen, the Netherlands; 3 Department of Critical Care, University Medical Center Groningen, University of Groningen, Hanzeplein 1, 9700 RB, Groningen, the Netherlands

## Abstract

**Background:**

Potassium depletion is common in hospitalized patients and can cause serious complications such as cardiac arrhythmias. In the intensive care unit (ICU) the majority of patients require potassium suppletion. However, there are no data regarding the optimal control target in critically ill patients. After open-heart surgery, patients have a strongly increased risk of atrial fibrillation or atrial flutter (AFF). In a novel trial design, we examined if in these patients different potassium control-targets within the normal range may have different effects on the incidence of AFF.

**Methods/Design:**

The "computer-driven Glucose and potassium Regulation program in Intensive care Patients with COMparison of PotASSium targets within normokalemic range (GRIP-COMPASS) trial" is a single-center prospective trial in which a total of 1200 patients are assigned to either a potassium control-target of 4.0 mmol/L or 4.5 mmol/L in consecutive alternating blocks of 50 patients each. Potassium levels are regulated by the computer-assisted potassium suppletion algorithm called GRIP-II (Glucose and potassium regulation for Intensive care Patients). Primary endpoint is the in-hospital incidence of AFF after cardiac surgery. Secondary endpoints are: in-hospital AFF in medical patients or patients after non-cardiac surgery, actually achieved potassium levels and their variation, electrolyte and glucose levels, potassium and insulin requirements, cumulative fluid balance, (ICU) length of stay, ICU mortality, hospital mortality and 90-day mortality.

**Discussion:**

The GRIP-COMPASS trial is the first controlled clinical trial to date that compares potassium targets. Other novel methodological elements of the study are that it is performed in ICU patients where both targets are within the normal range and that a computer-assisted potassium suppletion algorithm is used.

**Trial registration:**

NCT 01085071 at ClinicalTrials.gov

## Background

Potassium disorders occur frequently in hospitalized patients [1,2] and it has long been known that extreme potassium values can cause life-threatening complications, especially in critically ill patients [3,4]. However, the effects on outcome of less pronounced differences in the potassium concentration, that occur much more frequently, are not known.

As is the case with most laboratory parameters, plasma potassium values are considered as "normal" when they equal those observed in a healthy reference population. Although desirable levels for some laboratory parameters in critically patients may equal healthy reference levels, for others, such as hemoglobin, albumin or calcium, this is not the case [5-7]. Although potassium is administered to the majority of intensive care unit (ICU) patients, the optimal level in critically ill patients has never been investigated. There are no prospective trials concerning potassium regulation and only a few observational studies and reviews that describe the treatment once severe potassium disorders have developed [3,8,9]. Potassium has recently been involved in the debate on the disparate results of the Leuven [10] and NICE-SUGAR [11] trials that both compared glucose control targets, since the outcome of tight glucose control might be affected by potentially insufficiently controlled potassium levels [12].

Potassium regulation in the critically ill patient can be performed by titrated continuous potassium infusion. To avoid hyper- and hypokalemia, regular plasma potassium measurements and subsequent adjustments of the infusion rate are necessary [13-16]. To achieve two target values within a narrow range an effective potassium regulation protocol is mandatory. For safety and efficiency, computerized protocols appear to be superior over paper protocols [17-19]. In our ICU we have implemented an integrated computer-assisted program for both glucose and potassium regulation that is called GRIP-II (Glucose and potassium Regulation for Intensive care Patients) [20-22]. This program provides a recommendation on the potassium and insulin infusion rates and the time to the next potassium and glucose measurement. These recommendations are mainly based on the current potassium and glucose levels and analysis of their trend over time. In a before-after study comparing GRIP-II potassium control with physician-driven potassium control, potassium control improved with a significantly reduced number of hypokalemic and hyperkalemic events [20]. In the latter study the GRIP-II program aimed for a potassium level in the middle of the normal range of 3.5-5.0 mmol/L, i.e 4.3 mmol/L. However, it is not known what the effects would be when the target would be slightly different, i.e in normal-low or normal-high target range.

Potassium is the main intracellular cation of the body. The intracellular to extracellular potassium ratio affects both the fluid balance and the resting membrane potential. The reference plasma potassium ranges from 3.5 to 5.0 mmol/L. Levels below 3.0 mmol/L or above 6.0 mmol/L can cause serious symptoms of which cardiac arrhythmias are the most frequently observed [4]. After cardiac surgery (especially valvular surgery) postoperative atrial fibrillation is one of the most common complications with a reported incidence of 10-50%. It is associated with adverse outcome and despite new treatment strategies the incidence remains high [23-26]. After a potassium load, the intracellular compartment acts as the primary buffer. A potassium shift from extracellular to intracellular is caused by increased activity of the Na-K-ATPase pump, which can be directly stimulated by an increase in plasma potassium, insulin, catecholamines, aldosterone and alkalosis [27-29]. The most important organs in the homeostasis of potassium are the kidneys as they normally excrete or reabsorb potassium according to the overall potassium balance [30]. There is some evidence that a normal-high plasma potassium level is preferable in several cardiovascular states [31-33]. However, the optimal potassium level in the critically ill patient, who may be especially prone to the adverse effect of potassium derangements, has never been investigated prospectively. A cautious approach towards finding best target levels for potassium seems justified given the problematic discussions over optimal glucose targets in ICU patients [10,12]. A computer-assisted potassium regulation program such as GRIP-II provides the opportunity to perform a clinical trial to safely and efficiently compare two potassium targets within the normal range.

In this prospective phase 4 trial we will compare the effect of two different potassium control-target values that are both within the reference range of 3.5 - 5.0 mmol/L. In the NLP (Normal-Low Potassium) trial arm a normal-low potassium level of 4.0 mmol/L is aimed for, while in the NHP (Normal-High Potassium) trial arm a normal-high potassium level of 4.5 mmol/L is aimed for. We hypothesize that both physiological and pathophysiological differences may exist between the normal-low and the normal-high potassium control targets. In the study all patients are allocated to either the 4.0 or 4.5 mmol/L control target in alternating blocks of patients.

The GRIP-COMPASS trial thus constitutes a novel trial in a number of aspects. It is the first clinical trial to assess the effect of different potassium target levels. Also it is the first trial to compare two control-targets that are both within the reference range in critically ill patients. Furthermore this trial compares two treatment strategies that both are executed by a computer-based potassium suppletion algorithm.

## Methods/Design

**Figure 1:**
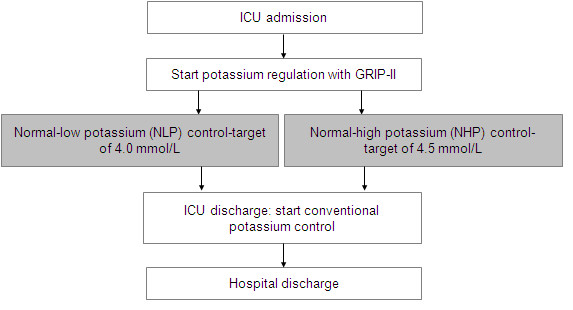
**The GRIP-COMPASS flow-chart**. ICU, intensive care unit; GRIP II, glucose and potassium regulation for intensive care patients.

**Figure 2:**
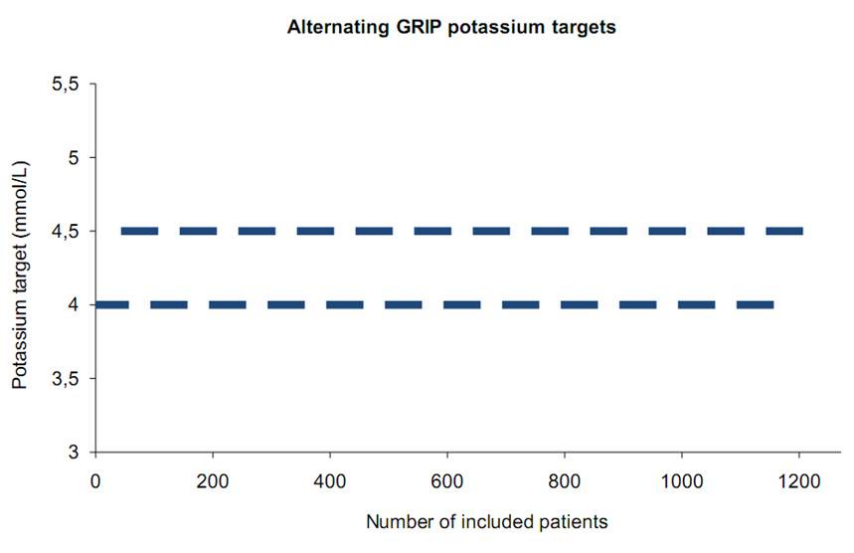
**Planned patient allocation scheme**. To create two comparable groups while reducing bias from various time-dependent system effects the GRIP computer is instructed to regularly alternate between the targets of 4.0 and 4.5 mmol/L. The goal is to include 50 patients in each block, where the precise timing of a block change may also depend on logistic factors.

The GRIP-COMPASS trial is a single-center, prospective trial with alternating blocks of patients with blinded evaluation of the primary endpoint. Twelve hundred patients are assigned to the NLP control-target of 4.0 mmol/L or the NHP control-target of 4.5 mmol/L at ICU admission (Figure 1). Allocation to an arm is alternated in blocks of approximately 50 patients until 1200 patients are included, resulting in 24 blocks (Figure 2). Informed consent for inclusion into one of the two study arms was waived by our institutional review board since in both the NLP arm and the NHP arm standard potassium treatment is involved with the only difference that the two therapeutic strategies aim at differing normal levels.

The study will be performed in a 14-bed thoracic-ICU (closed-format) of a tertiary university teaching hospital. The institutional medical ethics committee of the University Medical Center of Groningen approved the study (METc 2009/096). The trial protocol has been registered at ClinicalTrials.gov (NCT 01085071).

### Inclusion

All patients admitted to the thoracic ICU are considered eligible for inclusion. Excluded are those patients who do not have a central venous catheter or an enteral feeding tube because such patients are not entered into the GRIP-II program for potassium regulation since they cannot receive continuous potassium administration to the extent that is regularly necessary. Only rarely will patients be excluded for this reason. Immediately after admission a patient is assigned a potassium control-target that remains unchanged for that patient until discharge to the general ward. The general characteristics of the population admitted to the thoracic ICU are summarized in table 1. The majority of patients (~75%) are admitted after cardiac surgery, i.e. coronary artery bypass surgery (CABG) and/or valve repair.

**Table 1:** General characteristics of expected patient cohort.

	Thoracic ICU
	n = 500
Age (years), median (IQR)	66 (57-74)
Male sex	69%
APACHE II, median (IQR)	14 (10-18)
Reason for admission	
Cardiac surgery	75%
Cardiac arrest	9%
Other surgery	10%
Medical	5%
Miscellaneous	3%
post-operative AFF*	50%
In-hospital mortality	8%

*after cardiac surgery

Baseline characteristics of a recent cohort of 500 patients that were admitted to the thoracic ICU, to provide an impression of the expected patient population in the GRIP-COMPASS trial. The incidence of AFF was observed in a subgroup of 150 consecutive cardiac surgery patients. Abbreviations used in table: ICU intensive care unit; IQR interquartile range; APACHE II acute physiology and chronic health evaluation II score; AFF atrial fibrillation and atrial flutter.

### Potassium regulation

Potassium will be regulated by the computer-assisted decision support system called GRIP-II. This nurse-centered system for both potassium and glucose control was implemented at our ICU in 2004 to improve safety and efficiency. It is, to our knowledge, the first computer-assisted protocol for both glucose and potassium regulation. Both the design and results of both the glucose and potassium algorithm in a large and diverse cohort of ICU patients have been published previously [20-22]. GRIP-II provides a recommendation for the potassium and insulin infusion rate based the patients' potassium and glucose levels and the trend over time. It also gives an advice on the time-interval to the next potassium (and glucose) measurement and monitors if this measurement is performed on time. The average required number of point-of-care measurements per patient per day is 5.6, which is low compared to other computer-assisted glucose protocols [34,35].

Depending on the assigned group (NLP or NHP) GRIP-II will aim for a potassium control-target of 4.0 mmol/L or 4.5 mmol/L. The glucose target ranges are the same for both groups (4.0-7.5 mmol/L). Given the proximity of the targets of the NLP-arm and the NHP-arm that both lie comfortably within the reference range and since the spread in potassium levels actually achieved by GRIP-II was sufficiently small (median 4.3 with an interquartile range of 4.1-4.5 mmol/L) potassium levels for both trial arms are expected to stay within the reference range as well as would be the case without GRIP-II.

Both potassium and glucose are measured in arterial blood samples of 0.5 ml lithium-heparin anticoagulated blood using the ABL-800 series point-of-care analyser (Radiometer Copenhagen, Denmark) that is present on in the ICU. Potassium chloride is administered continuously by syringe pump either parenterally by central venous catheter or enterally. To minimize dosing errors it is administered in a "one-to-one" 1 mmol/ml solution. Potassium chloride suppletion in critically ill patients is efficient and safe in a dose-dependent and predictable way (independent of the use of diuretics or the kidney function) [15,16,36]. The maximum administration rate advised by GRIP-II is 20 mmol/hour. When a higher infusion rate is required, the attending physician can decide to administer more than 20 mmol/hour. Magnesium is supplemented at 30 mmol/day in patients after cardiac surgery [9]. In non-cardiac surgery patients, magnesium is only suppleted when total magnesium levels are <0.80 mmol/L.

### Primary endpoint

The primary endpoint is the incidence of in-hospital atrial fibrillation or atrial flutter (denoted by AFF), confirmed by a 12-lead electrocardiogram (ECG), in patients after cardiac surgery. The reference period over which this primary endpoint is determined is the first 7 days post-surgery, or until hospital discharge if the patient is discharged earlier. The presence of AFF is established by evaluation of all electrocardiograms by independent and qualified cardiologists who are blinded to the treatment allocations.

### Secondary endpoints

Secondary endpoints include:

- AFF that develops more than 7 days post-surgery and until hospital discharge.

- AFF in patients who do not undergo cardiac surgery during ICU stay, the first week after ICU discharge, until hospital discharge.

- The time course of the incidence of AFF (in cardiac-surgery patients and total patient group).

- The incidence of other serious arrhythmias that require immediate medical intervention (in cardiac-surgery patients and total patient group).

- Time that potassium is in the 3.5-5.0 mmol/L reference range during ICU-admission (in cardiac-surgery patients and total patient group).

- The occurrence of distinct hypokalemia (i.e potassium <2.8 mmol/L) and distinct hyperkalemia (i.e. potassium >6.0 mmol/L) in cardiac-surgery patients and the total patient group, also a safety endpoint.

- Level of glucose control and insulin requirements during ICU admission.

- Biochemical disturbances of electrolytes (sodium, magnesium and calcium), blood gas analysis, lactate and renal function (creatinine, urea) during hospital admission (total patient group).

- Cumulative fluid balance during ICU admission (patients admitted to the ICU for more than 5 days).

- ICU and hospital length of stay (in cardiac-surgery patients and total patient group).

- ICU-mortality and hospital mortality (in cardiac-surgery patients and total patient group, also a safety endpoint) as well as 90-day, and 1 year mortality.

In addition to the cardiac surgery group and the total patient group, the following subgroups will also be compared:

- Patients who underwent CABG with the use of cardiopulmonary bypass versus patients who underwent CABG without cardiopulmonary bypass.

- Patients with a history of AFF prior to surgery versus patients without a history of AFF prior to surgery.

### Data collection

Baseline patient characteristics are collected at ICU admission (age, sex, length, weight, medical history, medication use, reason of admission). If a patient undergoes (cardiac) surgery, data concerning the procedure and the duration of the procedure (and the time on cardiopulmonary bypass) are collected. For cardiac surgery patients the EuroSCORE model for operative risk stratification is calculated [37]. In non-cardiac surgery patients the APACHE II score is used [38].

This trial requires no specific tests or procedures that are not regularly performed. Daily laboratory tests and blood gas analysis (daily at the ICU, in the general ward only when deemed necessary by the physician) are all part of standard care. During ICU admission all patients are under continuous rhythm monitoring (3-lead) and a 12-lead ECG is done at admission (in cardiac-surgery patients also after 24 and 48 hours), and is repeated when arrhythmias or other complications occur or are suspected. At the general ward a the rhythm is checked regularly and a 12-lead ECG is performed at admission, when deemed necessary by the attending physician, and at discharge. When a patient is prone for severe arrhythmias, continuous monitoring by telemetry is continued at the general ward. All in-hospital ECGs of the included patients are evaluated by the physicians at the ICU and the general ward as well as by independent cardiologists who are blinded for the potassium control-targets.

Potassium and glucose control data (potassium and glucose levels, amount of potassium chloride and insulin administration, number of measurements per day) are automatically stored by the GRIP-II program. All other laboratory tests are stored in the hospital information system. ICU-parameters such as the amount of fluid administered, the use of vasoactive drugs, anti-arrhythmic drugs, diuresis, and the fluid balance are monitored and collected. Minor complications and major complications (e.g. death, stroke, myocardial infarction, organ failure and major bleeding complications) are recorded. Mortality is recorded for the ICU, during hospitalisation and at 90-days. Follow-up information will be obtained from hospital records and the central personal records database.

### Sample size calculation

Because the incidence of AFF in patients after cardiac surgery is the primary endpoint, the incidence recently measured in these patients and the expected potential difference in this incidence between the NLP and NHP groups were the main determinants of the power analysis. In a pilot study of 150 consecutive patients admitted after open-heart surgery (CABG and/or valve replacement) during a 3-month period, we observed that 50% developed postoperative AFF at the ICU or at the ward. To detect a 10% reduction in the incidence of AFF, with a two-sided level of significance of 5% and a power of 80%, 2 × 400 cardiac surgery patients should be included. Assuming that approximately 75% of the admitted patients will undergo cardiac surgery and with a 10% margin for excluded patients we arrived at a target number of 1200 consecutive patients admitted to the ICU to be included.

In a previous analysis of potassium regulation with GRIP-II, patients reached a stable potassium level within 12 hours with a mean ± standard deviation (SD) level of 4.25 ± 0.36 mmol/L [20]. When we assume that GRIP-II maintains this level of accuracy, then a significant difference in plasma potassium between the NLP and NHP groups will be found with less than 50 patients. Thus we do expect to easily detect very significant differences in the potassium levels between the NLP and NHP groups.

### Statistical analysis

To compare groups, the Student's t test (normally distributed variables), the Mann-Whitney U test (other continuous variables) or Fisher's exact test (categorical variables) will be used when appropriate. The level of potassium control will be expressed as the time within the normokalemic range (3.5 - 5.0 mmol/L) divided by the total time spent on the ICU.

In multivariate analysis we will investigate the interaction with glucose, the amount of administered insulin, sodium and magnesium. A two-sided P value of <0.05 will be considered significant. The Statistical package for the social sciences (SPSS) version 16.0 will be used for all statistical analysis.

## Conclusion

The GRIP-COMPASS trial is a single-center, prospective, controlled trial with an alternating patient design to compare the effect of two different potassium control-targets that are both within the normal range in 1200 ICU patients. A validated computer-assisted potassium regulation algorithm has an indispensable role in carrying out this large trial. The primary outcome measure will be the incidence of atrial fibrillation and flutter in patients after cardiac surgery.

## List of abbreviations used

ICU: intensive care unit; GRIP-II: glucose and potassium regulation for intensive care patients; NLP: normal-low potassium; NHP: normal-high potassium; CABG: coronary artery bypass surgery; ECG: electrocardiogram; AFF: atrial fibrillation/atrial flutter; EuroSCORE: European system for cardiac operative risk evaluation; APACHE-II: score acute physiology and chronic health evaluation - II score.

## Competing interests

The authors declare that they have no competing interests.

## Authors' contributions

MH, FZ, IvdH and MN participated in study design. MH, MV and MN participated in data acquisition and analysis. MV and MN participated in programming the computer software. MH and MN participated in drafting and interpretation of the manuscript. IvdH, AOL, JvdM, FI and FZ participated in interpretation of the manuscript. All authors read and approved the final manuscript.

## Pre-publication history

The pre-publication history for this paper can be accessed here:

http://www.biomedcentral.com/1471-2253/10/23/prepub

## References

[B1] Acker CG Johnson JP Palevsky PM Greenberg A Hyperkalemia in hospitalized patients: causes, adequacy of treatment, and results of an attempt to improve physician compliance with published therapy guidelines Arch Intern Med 1998 158 917 924 10.1001/archinte.158.8.917 9570179

[B2] Paltiel O Salakhov E Ronen I Berg D Israeli A Management of severe hypokalemia in hospitalized patients: a study of quality of care based on computerized databases Arch Intern Med 2001 161 1089 1095 10.1001/archinte.161.8.1089 11322843

[B3] Weisberg LS Szerlip HM Cox M Disorders of potassium homeostasis in critically ill patients Crit Care Clin 1987 3 835 854 3332226

[B4] Schwartz AB Potassium-related cardiac arrhythmias and their treatment Angiology 1978 29 194 205 10.1177/000331977802900302 646184

[B5] Hebert PC Wells G Blajchman MA Marshall J Martin C Pagliarello G A multicenter, randomized, controlled clinical trial of transfusion requirements in critical care. Transfusion Requirements in Critical Care Investigators, Canadian Critical Care Trials Group N Engl J Med 1999 340 409 417 10.1056/NEJM199902113400601 9971864

[B6] Dellinger RP Levy MM Carlet JM Bion J Parker MM Jaeschke R Surviving Sepsis Campaign: international guidelines for management of severe sepsis and septic shock: 2008 Crit Care Med 2008 36 296 327 10.1097/01.CCM.0000298158.12101.41 18158437

[B7] Desai TK Carlson RW Geheb MA Prevalence and clinical implications of hypocalcemia in acutely ill patients in a medical intensive care setting Am J Med 1988 84 209 214 10.1016/0002-9343(88)90415-9 3407650

[B8] Gennari FJ Disorders of potassium homeostasis. Hypokalemia and hyperkalemia Crit Care Clin 2002 18 273 88 vi 10.1016/S0749-0704(01)00009-4 12053834

[B9] Hamill-Ruth RJ McGory R Magnesium repletion and its effect on potassium homeostasis in critically ill adults: results of a double-blind, randomized, controlled trial Crit Care Med 1996 24 38 45 10.1097/00003246-199601000-00009 8565536

[B10] van den Berghe G Wouters P Weekers F Verwaest C Bruyninckx F Schetz M Intensive insulin therapy in the critically ill patients N Engl J Med 2001 345 1359 1367 10.1056/NEJMoa011300 11794168

[B11] Finfer S Chittock DR Su SY Blair D Foster D Dhingra V Intensive versus conventional glucose control in critically ill patients N Engl J Med 2009 360 1283 1297 10.1056/NEJMoa0810625 19318384

[B12] van den Berghe G Schetz M Vlasselaers D Hermans G Wilmer A Bouillon R Clinical review: Intensive insulin therapy in critically ill patients: NICE-SUGAR or Leuven blood glucose target? J Clin Endocrinol Metab 2009 94 3163 3170 10.1210/jc.2009-0663 19531590

[B13] Kanji Z Jung K Evaluation of an electrolyte replacement protocol in an adult intensive care unit: a retrospective before and after analysis Intensive Crit Care Nurs 2009 25 181 189 10.1016/j.iccn.2009.03.004 19398203

[B14] Cohn JN Kowey PR Whelton PK Prisant LM New guidelines for potassium replacement in clinical practice: a contemporary review by the National Council on Potassium in Clinical Practice Arch Intern Med 2000 160 2429 2436 10.1001/archinte.160.16.2429 10979053

[B15] Hamill RJ Robinson LM Wexler HR Moote C Efficacy and safety of potassium infusion therapy in hypokalemic critically ill patients Crit Care Med 1991 19 694 699 10.1097/00003246-199105000-00016 2026032

[B16] Kruse JA Carlson RW Rapid correction of hypokalemia using concentrated intravenous potassium chloride infusions Arch Intern Med 1990 150 613 617 10.1001/archinte.150.3.613 2310280

[B17] Paltiel O Gordon L Berg D Israeli A Effect of a computerized alert on the management of hypokalemia in hospitalized patients Arch Intern Med 2003 163 200 204 10.1001/archinte.163.2.200 12546610

[B18] Boord JB Sharifi M Greevy RA Griffin MR Lee VK Webb TA Computer-based insulin infusion protocol improves glycemia control over manual protocol J Am Med Inform Assoc 2007 14 278 287 10.1197/jamia.M2292 17329722 PMC2244871

[B19] Hemstreet BA Stolpman N Badesch DB May SK McCollum M Potassium and phosphorus repletion in hospitalized patients: implications for clinical practice and the potential use of healthcare information technology to improve prescribing and patient safety Curr Med Res Opin 2006 22 2449 2455 10.1185/030079906X148463 17257459

[B20] Hoekstra M Vogelzang M Drost JT Janse M Loef BG van der Horst IC Implementation and evaluation of a nurse-centered computerized potassium regulation protocol in the intensive care unit - a before and after analysis BMC Med Inform Decis Mak 2010 10 5 10.1186/1472-6947-10-5 20100342 PMC2826292

[B21] Vogelzang M Zijlstra F Nijsten MW Design and implementation of GRIP: a computerized glucose control system at a surgical intensive care unit BMC Med Inform Decis Mak 2005 5 38 10.1186/1472-6947-5-38 16359559 PMC1334184

[B22] Vogelzang M Loef BG Regtien JG van der Horst IC van Assen H Zijlstra F Computer-assisted glucose control in critically ill patients Intensive Care Med 2008 34 1421 1427 10.1007/s00134-008-1091-y 18389221 PMC2491417

[B23] El Chami MF Kilgo P Thourani V Lattouf OM Delurgio DB Guyton RA New-onset atrial fibrillation predicts long-term mortality after coronary artery bypass graft J Am Coll Cardiol 2010 55 1370 1376 10.1016/j.jacc.2009.10.058 20338499

[B24] Daoud EG Strickberger SA Man KC Goyal R Deeb GM Bolling SF Preoperative amiodarone as prophylaxis against atrial fibrillation after heart surgery N Engl J Med 1997 337 1785 1791 10.1056/NEJM199712183372501 9400034

[B25] Shepherd J Jones J Frampton GK Tanajewski L Turner D Price A Intravenous magnesium sulphate and sotalol for prevention of atrial fibrillation after coronary artery bypass surgery: a systematic review and economic evaluation Health Technol Assess 2008 12 iii 95 10.3310/hta12280 18547499

[B26] Chen WT Krishnan GM Sood N Kluger J Coleman CI Effect of statins on atrial fibrillation after cardiac surgery: A duration- and dose-response meta-analysis J Thorac Cardiovasc Surg 2010 20381820 10.1016/j.jtcvs.2010.02.042

[B27] Clausen T Everts ME Regulation of the Na,K-pump in skeletal muscle Kidney Int 1989 35 1 13 10.1038/ki.1989.1 2540370

[B28] Giebisch G Krapf R Wagner C Renal and extrarenal regulation of potassium Kidney Int 2007 72 397 410 10.1038/sj.ki.5002288 17568786

[B29] Zierler K Rogus EM Scherer RW Wu FS Insulin action on membrane potential and glucose uptake: effects of high potassium Am J Physiol 1985 249 E17 E25 3893153 10.1152/ajpendo.1985.249.1.E17

[B30] Giebisch G Wang W Potassium transport: from clearance to channels and pumps Kidney Int 1996 49 1624 1631 10.1038/ki.1996.236 8743466

[B31] Macdonald JE Struthers AD What is the optimal serum potassium level in cardiovascular patients? J Am Coll Cardiol 2004 43 155 161 10.1016/j.jacc.2003.06.021 14736430

[B32] He FJ MacGregor GA Fortnightly review: Beneficial effects of potassium BMJ 2001 323 497 501 10.1136/bmj.323.7311.497 11532846 PMC1121081

[B33] Schulman M Narins RG Hypokalemia and cardiovascular disease Am J Cardiol 1990 65 4E 9E 10.1016/0002-9149(90)90244-U 2178377

[B34] Hoekstra M Vogelzang M Verbitskiy E Nijsten MW Health technology assessment review: Computerized glucose regulation in the intensive care unit - how to create artificial control Crit Care 2009 13 223 10.1186/cc8023 19849827 PMC2784347

[B35] Hoekstra M Vogelzang M Verbitskiy E Nijsten MW Hourly measurements not required for safe and effective glycemic control in the critically ill patient Crit Care 2010 14 404 10.1186/cc8190 20156329 PMC2875483

[B36] Kruse JA Clark VL Carlson RW Geheb MA Concentrated potassium chloride infusions in critically ill patients with hypokalemia J Clin Pharmacol 1994 34 1077 1082 7876399 10.1002/j.1552-4604.1994.tb01984.x

[B37] Nashef SA Roques F Michel P Gauducheau E Lemeshow S Salamon R European system for cardiac operative risk evaluation (EuroSCORE) Eur J Cardiothorac Surg 1999 16 9 13 10.1016/S1010-7940(99)00134-7 10456395

[B38] Knaus WA Draper EA Wagner DP Zimmerman JE APACHE II: a severity of disease classification system Crit Care Med 1985 13 818 829 10.1097/00003246-198510000-00009 3928249

